# Effects of Dietary Fish Oil on the Depletion of Carcinogenic PAH-DNA Adduct Levels in the Liver of B6C3F1 Mouse

**DOI:** 10.1371/journal.pone.0026589

**Published:** 2011-10-31

**Authors:** Guo-Dong Zhou, Huiping Zhu, Tracie D. Phillips, Jianbo Wang, Shi-Zhou Wang, Fen Wang, Brad A. Amendt, Xanthi I. Couroucli, Kirby C. Donnelly, Bhagavatula Moorthy

**Affiliations:** 1 Institute of Biosciences and Technology, Texas A&M University System Health Science Center, Houston, Texas, United States of America; 2 School of Rural Public Health, Texas A&M University System Health Science Center, College Station, Texas, United States of America; 3 Dell Pediatric Research Institute, University of Texas, Austin, Texas, United States of America; 4 Department of Pediatrics, Baylor College of Medicine, Houston, Texas, United States of America; IIT Research Institute, United States of America

## Abstract

Many carcinogenic polycyclic aromatic hydrocarbons (PAHs) and their metabolites can bind covalently to DNA. Carcinogen-DNA adducts may lead to mutations in critical genes, eventually leading to cancer. In this study we report that fish oil (FO) blocks the formation of DNA adducts by detoxification of PAHs. B6C3F1 male mice were fed a FO or corn oil (CO) diet for 30 days. The animals were then treated with seven carcinogenic PAHs including benzo(a)pyrene (BaP) with one of two doses via a single intraperitoneal injection. Animals were terminated at 1, 3, or 7 d after treatment. The levels of DNA adducts were analyzed by the ^32^P-postlabeling assay. Our results showed that the levels of total hepatic DNA adducts were significantly decreased in FO groups compared to CO groups with an exception of low PAH dose at 3 d (P = 0.067). Total adduct levels in the high dose PAH groups were 41.36±6.48 (Mean±SEM) and 78.72±8.03 in 10^9^ nucleotides (P = 0.011), respectively, for the FO and CO groups at 7 d. Animals treated with the low dose (2.5 fold lower) PAHs displayed similar trends. Total adduct levels were 12.21±2.33 in the FO group and 24.07±1.99 in the CO group, P = 0.008. BPDE-dG adduct values at 7 d after treatment of high dose PAHs were 32.34±1.94 (CO group) and 21.82±3.37 (FO group) in 10^9^ nucleotides with P value being 0.035. Low dose groups showed similar trends for BPDE-dG adduct in the two diet groups. FO significantly enhanced gene expression of *Cyp1a1* in both the high and low dose PAH groups. *Gstt1* at low dose of PAHs showed high levels in FO compared to CO groups with P values being 0.014. Histological observations indicated that FO played a hepatoprotective role during the early stages. Our results suggest that FO has a potential to be developed as a cancer chemopreventive agent.

## Introduction

According to the World Health Organization, cancer is a leading cause of death worldwide. It accounted for 7.6 million deaths (around 13% of all deaths worldwide) in 2008 [1]. In United States, a total of 1,529,560 new cancer cases and 569,490 deaths from cancer were projected to occur in the United States in 2010 [2]. Epidemiological and experimental data show that most cancers are caused by environmental factors, including exposure to complex mixtures such as carcinogenic polycyclic aromatic hydrocarbons (PAHs) from cigarette smoke, air and water pollution [3]. Several PAHs have been listed by the U.S. EPA as probable human carcinogens [4]. PAHs exhibit their biological effects through metabolic activation by cytochrome P450 (CYP) enzymes to electrophilic species that are capable of reacting with nucleophilic sites of DNA to form adducts. Such pre-mutagenic lesions play essential roles in the initiation stage of carcinogenesis [5], [6].

A prospective cohort study, reported by Tang et al [7], showed that healthy current smokers who had elevated levels of aromatic DNA adducts in white blood cells were approximately three times more likely to be diagnosed with lung cancer one to thirteen years later than current smokers with lower adduct levels. Bak et al. [8] obtained similar epidemiological results in Denmark. Animal (B6C3F1 male mice) experiments have also shown significant positive correlations between levels of DNA adducts induced by BaP or complex PAHs mixtures at 1, 3, and 7 days after treatment and tumor incidence observed after 10 months [9]. Therefore, decreases in carcinogen-DNA adduct levels by appropriate approaches, including using chemopreventive agents, is expected to lower tumor yields. Since most cancers are still not curable, cancer prevention, especially in initiation phase of carcinogenesis is much more desirable than that during the phases of promotion and progression.

Recently, natural chemopreventive agents have received great attention for cancer prevention because of their various health benefits, such as lack of toxicity, and lesser side effects [10]. Chemopreventive agents may inhibit adduct formation or remove adducted nuleotides and damaged cells through several pathways: 1) metabolic inactivation of chemical carcinogens [11], [12]; 2) enhancement of DNA repair [13]; and 3) induction of cellular apoptosis [14], [15]. Previous experiments have shown that dietary fish oil (FO) with major components of n-3 fatty acids decreases oxidative DNA lesions by enhancing apoptosis in rat colon [16], [17]. It has been reported that oxidation of n-3 fatty acids can induce Nrf2-based antioxidant and phase II detoxification defense systems [18]. Mernitz et al reported that FO inhibits NNK-induced lung carcinogenesis in the A/J mouse [19]. It has also been reported that dietary FO suppresses tumor growth and metastasis [20]. Fat-1 transgenic mice (convert n-6 to n-3 fatty acids) [21] displayed a decrease in tumor formation in the diethylnitrosamine induced liver tumor model compared to wild-type littermates [22]. Liver Cox-2 expression was markedly lower in these fat-1 mice [22]. Lim et al reported that n-3 fatty acids inhibit hepatocellular carcinoma cell growth through blocking β-catenin and Cox-2 [23].

In this investigation, we have tested the hypothesis that levels of carcinogenic PAH-DNA adducts can be diminished by dietary FO. The mechanisms of attenuation of DNA adducts by dietary FO have been also investigated.

## Materials and Methods

### Ethics Statement

Animal experiments were carried out in strict accordance with the principles and procedures of the Guide for the Care and Use of Laboratory Animals. The protocol was approved by the Institutional Animal Care and Use Committee, Texas A&M University System Health Science Center (Protocol Number: 06001-R1).

### Chemicals

Benzo(a)pyrene (BaP), benz(a)anthracene (BA), chrysene, benzo(b)fluoranthene (BbF), benzo(k)fluoranthene (BkF), and dibenz(a,h)anthracene (DBA) were obtained from Sigma-Aldrich (St. Louis, MO). Indeno(1,2,3-c,d)pyrene (IP) was purchased from Absolute Standards (Hamden, CT). Materials for DNA extraction [24], [25], [26], [27], RNA extraction [28], and ^32^postlabeling analysis [24], [29], [30] have been reported previously.

### Animals

B6C3F1 male mice (4–5 weeks) were obtained from Harlan Sprague Dawley (Houston). A total of 70 animals were initially housed in the Program for Animal Resources of the Institute of Biosciences and Technology, Texas A&M University Syetem.

### Diets

FO and CO diets were purchased from Dytes Inc. (Bethlehem, PA). All diets contained 15% lipid by weight (Table 1). Menhaden fish oil was provided by Omega Protein Inc. (Houston, TX). The fatty acid compositions of FO and CO are listed in Table 2. In order to meet essential fatty acid requirements, the FO diet contained 3.5 g of corn oil/100 g of diet [31], [32]. To prevent formation of oxidized lipids, the diet was stored at −20°C in the dark. The FO and CO diets contained identical levels of α-tocopherol (0.15%), γ-tocopherol (0.1%) and tertiarybutylhydroquinine (0.025%) as antioxidants.

**Table 1:** Diet composition.

Ingredients	Fish Oil Diet (%)	Corn Oil Diet (%)
Dextrose	51.05	51.05
Casein	22.35	22.35
DL-Methionine	0.34	0.34
Corn Oil	3.50	15.00
Fish Oil (Menhaden)	11.50	0
Mineral mix, AIN-76, Harlan Teklad	3.91	3.91
Vitamin mix, AIN 76-A, Harlan Teklad	1.12	1.12
Choline bitartrate	0.22	0.22
Pectin	6.00	6.00

**Table 2:** Fatty acids in fish oil and corn oil.

Fish Oil						Corn Oil	
Fatty acid	Weight (%)	Fatty acid	Weight (%)	Fatty acid	Weight (%)	Fatty acid	Weight (%)
14∶0	9.0	18∶0	2.8	20∶5 (n-3)	15.5	14∶0	Trace
15∶0	0.7	18∶1	11.4	21∶5 (n-3)	0.8	16∶0	10.8
16∶0	17.1	18∶2 (n-6)	1.5	22∶1	0.5	16∶1	Trace
16∶1	12.5	18∶3*	1.4	22∶4 (n-6)	0.4	18∶0	2.1
16∶2	1.7	18∶4 (n-3)	3.5	22∶5 (n-3)	2.4	18∶1	26.5
16∶3	1.7	20∶0	0.2	22∶6 (n-3)	9.1	18∶2 (n-6)	60.0
16∶4 (n-3)	1.8	20∶1	1.6	24∶1	0.1	18∶3***	0.6
17∶0	0.9	20∶3 (n-6)	0.4	Unknown	0.2	Total	100
17∶1	0.5	20∶4**	2.3	Total	100		

*n-3 = 0.99%, n-6 = 0.41%;

**n-3 = 1.4%, n-6 = 0.9%;

***n-3/n-6.

### Animal experiments

Mice were housed in cages in a temperature and humidity controlled facility with a daily photoperiod of 12 hours light and 12 hours dark. Animals were acclimatized for a week to the new environment during which the mice consumed a standard pelleted diet, and the mice were randomly allocated to one of the two experimental diet groups. Mice had free access to water and fresh diet was provided in clean food bowls daily. After 30 days of consuming the experimental diets (FO or CO), 35 animals were randomized into 7 groups of 5 mice each for each diet. Then 15 mice for each diet were intraperitioneally (ip) injected with a mixture of high dose PAHs, i.e. BaP, 1.72 µg; BA, 5.25 µg; chrysene, 5.25 µg; BbF, 2.15 µg; BkF, 1.33 µg; DBA, 0.11 µg and IP, 0.67 µg for 20 g body weight. Another 15 mice for each diet received low dose PAHs. These chemicals and their concentrations were similar to the residues extracted from contaminated soils of SuperFund Sites [9]. The concentrations of low dose PAHs were proportionally reduced by 2.5 fold from high dose PAHs mixture. These chemicals were dissolved in dimethyl sulfoxide (DMSO):corn oil (50∶50). Vehicle volume for ip injection was 7 µl per g body weight. Animals were fed same diet after treatment and were euthanized by CO_2_ asphyxiation and cervical dislocation at 1, 3, and 7 d after the PAH treatment. Five mice served as a control for each diet and therefore, were injected with vehicle. Liver tissues were collected and were frozen in liquid nitrogen immediately and then stored at −80°C until extractions of DNA and RNA. Part of each tissue was fixed in paraformaldehyde (4%) for histological examination.

### Analyses of DNA adducts

DNA extraction and ^32^P-postlabeling analyses were performed as in previous experiments [32]. DNA was isolated by solvent extraction combined with enzymatic digestion of protein and RNA [26], [27] and stored at −80°C until analysis. The nuclease P1-enhanced bisphosphate version of the ^32^P-postlabeling method [24] was used for analysis, with modifications of the chromatographic conditions [33]. Briefly, DNA (10 µg) was enzymatically degraded to normal (Np) and modified (Xp) deoxyribonucleoside 3′-monophosphates with micrococcal nuclease and spleen phosphodiesterase at pH 6.0 and 37°C for 3.5 h. After treatment of the mixture with nuclease P1 to convert normal nucleotides to nucleosides, modified nucleotides (Xp) were converted to 5′-^32^P-labeled deoxyribonucleoside 3′,5′-bisphosphates (pXp) by incubation with carrier-free [γ-^32^P]ATP and polynucleotide kinase.

Radioactively labeled digests were applied to modified polyethyleneimine (PEI)-cellulose thin layers and chromatographed overnight (15–16 h) with 2.3 M sodium phosphate, pH 5.75 (D1), to purify bulky adducts. Labeled PAH-adducts retained in the lower (L, 2.8×1.0 cm) part of the D1 chromatogram were, after brief autoradiography on Cronex 4 X-ray film, each contact-transferred to individual acceptor sheets and resolved by two-dimensional thin-layer chromatography (TLC). The bulky DNA adducts were separated with 3.82 M lithium formate, 6.75 M urea, pH 3.35 and 0.72 M sodium phosphate, 0.45 M Tris–HCl, 7.65 M urea, pH 8.2 in the first (D3) and second (D4) dimensions, respectively. ^32^P-labeled DNA adducts were visualized by screen-enhanced autoradiography at −80°C using Kodak XAR-5 film or with the aid of an InstantImager (Packard Instruments) [25]. Appropriate blank count rates were automatically subtracted by the instrument from sample values. The extent of covalent DNA modification was calculated from corrected sample count rates. Quantitative data represented minimum estimates because 100% recovery presumably was not achieved. Statistical analysis of the data from individual mice was performed by using the unpaired and paired Student's *t*-tests [34].

### Quantitative reverse-transcription PCR

TaqMan® Gene Expression Assays were used to determine gene expression changes for selected detoxification genes. Gene-specific probes and primer sets were purchased from Applied Biosystems (Foster City, CA). The assays were performed according to manufacturer's protocol on an ABI PRISM® 7900 HT Sequence Detection System (Applied Biosystems, Foster City, CA). Data were analyzed using SDS software v2.1 (Applied Biosystems, Foster City, CA). Mouse *Gapdh* gene was used as house-keeping control for quantitative RT-PCR because it exhibited consistent normalized intensity across all arrays. Relative standard curve method was used to generate quantitative values. Each reaction was replicated three times and the normalized mean value was used in the final comparisons. In order to compare the levels of gene expression in liver between two diet groups with an unpaired T-test, Ct values were converted by the formula: 2∧(−(Ct value − β-actin value − a constant)).

### Histological examination

The animals were euthanized by CO_2_ asphyxiation and cervical dislocation. The livers were removed for various analyses. Part of each tissue was fixed in 4% paraformaldehyde, following which the samples were embedded in paraffin and cut into 5 µm thick sections. The tissue section on glass slide was stained with hematoxylin and eosin (H&E).

## Results

### Diet consumption and body weights

B6C3F1 male mice were fed dietary FO or CO for 30 days before treatment with PAH mixture. During the experiment, diet consumption was measured daily. Average daily diet consumption was 2.96±0.034 g (mean ± SEM) and 3.01±0.028 g per mouse for FO and CO groups, respectively. The differences were not significant (P>0.05). The body weights were determined weekly. Dietary FO did not significantly affect the body weights compared to CO group (Table 3).

**Table 3:** Mouse body weights.

Time	Corn Oil		Fish Oil		P
	Mean	SEM	Mean	SEM	
Day 0	21.53	0.19	21.53	0.16	>0.05
Day 7	23.89	0.22	23.86	0.20	>0.05
Day 14	26.09	0.25	25.94	0.21	>0.05
Day 21	28.02	0.25	28.19	0.25	>0.05
Day 28	29.82	0.31	30.49	0.26	>0.05

n = 35.

### Effects of dietary fish oil on PAH-DNA adducts

**Figure 1:**
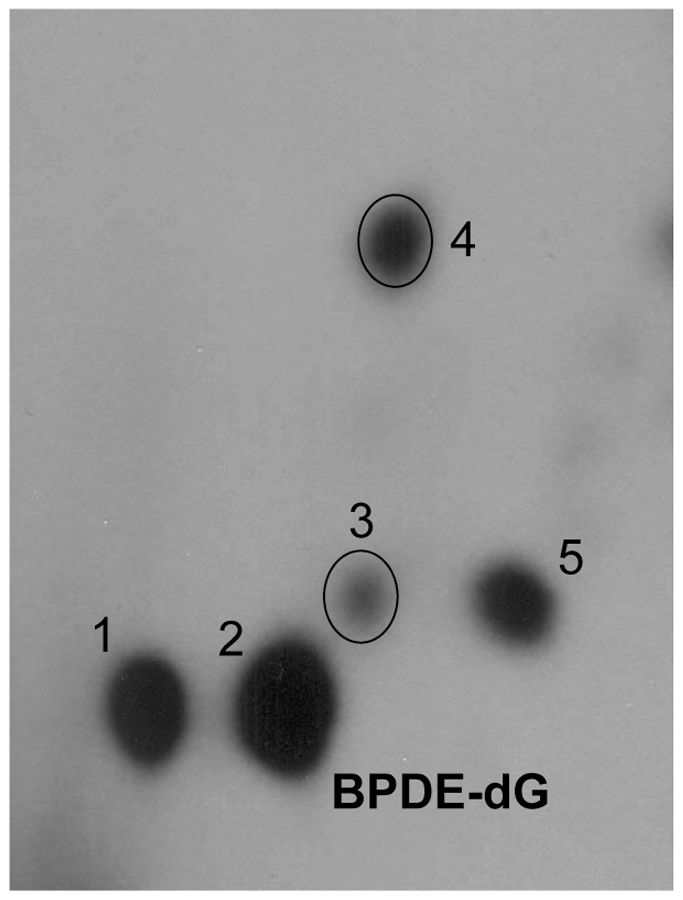
Typical representative pattern of ^32^P-postlabeled PAH-DNA adducts in mouse liver. Mice, fed with dietary FO, were treated with high dose of PAH mixture. Animals were terminated at 7 days after treatment.

Liver DNA from mice fed with dietary FO or CO exhibited qualitatively similar profiles of DNA adducts (Figure 1). This typical pattern displayed that BPDE-dG [35] was the major adduct.

**Figure 2:**
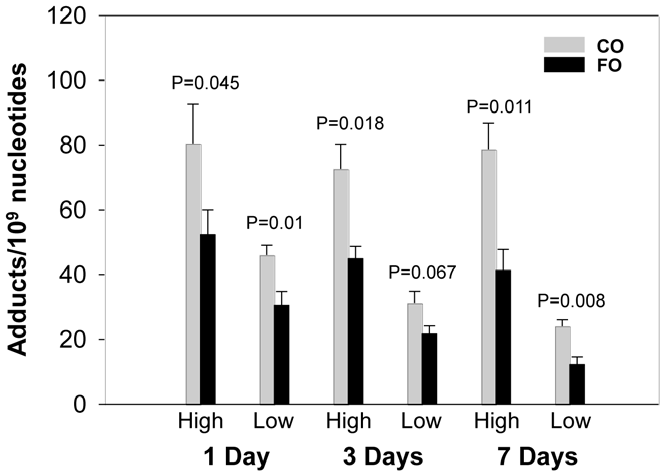
Comparison of the levels of total DNA adducts in liver of mice between two diet groups. Mice were treated with high or low dose of PAH mixtures. Animals were terminated at 1, 3 or 7 days after treatment. Overall, dietary FO decreased the levels of PAH adducts compared to CO groups. Except for low PAH dose at 3 days, the differences of adduct values between two diet groups were significant. Mean ± SEM, n = 4.

**Figure 3:**
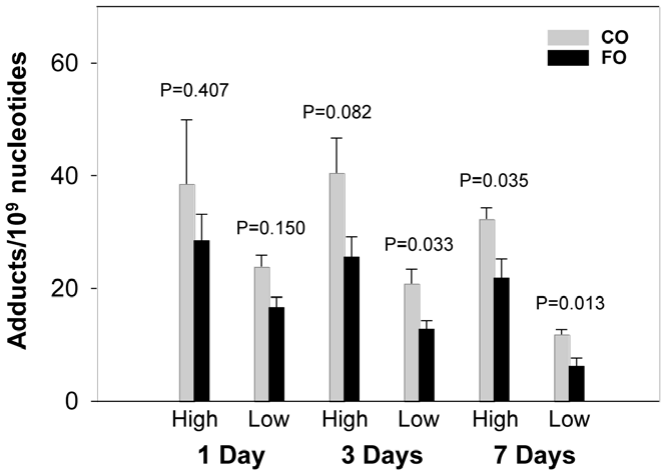
Comparison of the levels of BPDE-dG adduct in liver of mice between the two diet groups. FO significantly reduced the levels of BPDE-dG adducts at 3 (low PAH dose only) or 7 day time points. For animal treatment information, please see Figure 2. Mean ± SEM, n = 4.

Hepatic DNA adducts were formed within 24 h after treatment of PAH mixture. High levels of PAH adducts persisted from 24 h till 7 d after treatment. Overall, DNA adducts displayed the highest levels at 1 d time point. The levels of DNA adducts were then decreased at the 3 and 7 d time points in both dietary groups (Figures 2 and 3). The levels of BPDE-dG and total DNA adducts (spots 1–5, Figure 1) in livers of mice were much higher in CO groups compared to corresponding FO groups at all time points. Dietary FO decreased levels of total PAH adducts by 30–49% (Figure 2). BPDE-dG adduct displayed similar results and the adduct levels in FO groups were attenuated by 26–47% (Figure 3).

Although FO groups showed lower levels of DNA adducts at all time points in both PAH doses compared to CO groups, the significant differences in both BPDE-dG and total adduct levels were only observed at 7 d time point. At high PAH dose, the values of total hepatic DNA adducts were 78.72±8.03 (mean±SEM) and 41.35±6.48 in 10^9^ nucleotides for CO and FO groups, respectively. The P value was 0.011 (Figure 2). The levels of BPDE-dG adducts were 32.34±1.94 (CO group) and 21.82±3.37 (FO group) in 10^9^ nucleotides with P value being 0.035 (Figure 3). Low dose PAH groups displayed similar trends for both BPDE-dG and total adducts. Interestingly, at 1 d time point, total DNA adducts displayed significant differences between CO and FO groups (Figure 2). However, no significant differences of BPDE-dG levels were observed between two diet groups, although FO showed trends of lower adduct levels compared to CO. At 3 d time point, total adduct levels at high PAH dose group, but not low dose, displayed significant difference between two diet groups. The levels of DNA adducts were 72.58±7.71 for CO group and 44.99±3.82 for FO group (P = 0.018). FO decreased level of DNA adducts by 38% (Figure 2). BPDE-dG adduct at low PAH dose group, but not high dose, showed similar results with value of adducts being 20.90±2.53 and 12.73±1.55 for CO and FO groups, respectively. P value was 0.033 (Figure 3). Overall, BPDE-dG and total adducts were diminished by dietary FO in both high and low dose PHA groups (Figures 2 and 3). Taking all the groups of different treatments and time points together, dietary FO showed tremendous effects on the formation of DNA adducts (Table 4). Paired t-test displayed very significant lowering of PAH adducts in FO groups compared with CO groups. P values were 0.0008 and 0.0048 for BPDE-dG and total adducts, respectively.

**Table 4:** Comparison of levels of DNA adducts in livers of male mice treated with mixtures of carcinogenic PAHs between two diets (adducts in 10^9^ nucleotides).

Time	Chemical	Dose	BPDE-dG Adduct			Total DNA adducts		
			CO	FO	P	CO	FO	P
1 day	PAHs	High	38.55	28.48	0.0008	80.49	52.35	0.0048
		Low	23.89	16.59		46.07	30.63	
3 day	PAHs	High	40.48	25.56		72.58	44.99	
		Low	20.90	12.73		31.25	21.76	
7 day	PAHs	High	32.34	21.82		78.72	41.35	
		Low	11.86	6.23		24.07	12.21	

### Effects of dietary fish oil on gene expression of phase I and II enzymes

In order to understand the mechanisms that may contribute to the decreased levels of DNA adducts, including chemical detoxification, DNA repair and apoptosis, which are enhanced by FO, gene expression was analyzed by Real-time RT-PCR. Gene expression of phase I and II enzymes *Cyp1a1*, *Cyp1b1*, *Gstt1*, and *Gstm1* in hepatic RNA of mice at 7 d time point was determined by TaqMan® Gene Expression assays. FO significantly enhanced activities of *Cyp1a1* (ΔΔ Ct method) compared to CO in both low and high doses of PAH treated groups (Table 5). *Gstt1* expression was only enhanced by FO at low dose of PAH group. In contrast, *Cyp1b1* and *Gstm1* did not significantly change as dramatically as *Cyp1a1* and *Gstt1* between the two diet groups. DNA repair and apoptosis related genes, *Dclre1a*, *Ercc1*, and *Casp3*, in the liver of 7 d time point mice were also determined by Real-time RT-PCR. There were no significant differences in hepatic expression of these genes between the two diet groups (data not shown). Gene expression of Gstt1 and Gstm1 was also analyzed for 1 and 3 d time points. However, no significant differences were observed between CO and FO.

**Table 5:** Effects of dietary fish oil on hepatic gene expression of some metabolic enzymes.

Gene	Group	Normalized ΔΔ Ct values		P
		CO (Mean±SEM)	FO (Mean±SEM)	
Cyp1a1*	DMSO	5.38±0.36	5.28±0.13	0.903
	HD PAHs	2.69±0.07	6.32±0.31	<0.001
	LD PAHs	3.52±0.16	4.41±0.12	0.038
Cyp1b1*	DMSO	7.66±0.60	7.73±0.56	0.967
	HD PAHs	8.65±0.59	8.58±0.45	0.963
	LD PAHs	8.19±0.54	9.88±0.46	0.232
Gstt1**	DMSO	8.20±1.19	18.38±3.63	0.037
	HD PAHs	21.03±2.60	27.05±1.69	0.100
	LD PAHs	15.06±2.25	24.58±1.61	0.014
Gstm1**	DMSO	2.40±0.26	2.65±0.18	0.460
	HD PAHs	7.37±.11	8.24±0.61	0.517
	LD PAHs	2.71±0.29	2.74±0.33	0.947

*2∧(−(Ct value − β-actin value − 15));

**2∧(−(Ct value − β-actin value − 3));

n = 4.

### Hepatic toxicity induced by PAHs

**Figure 4:**
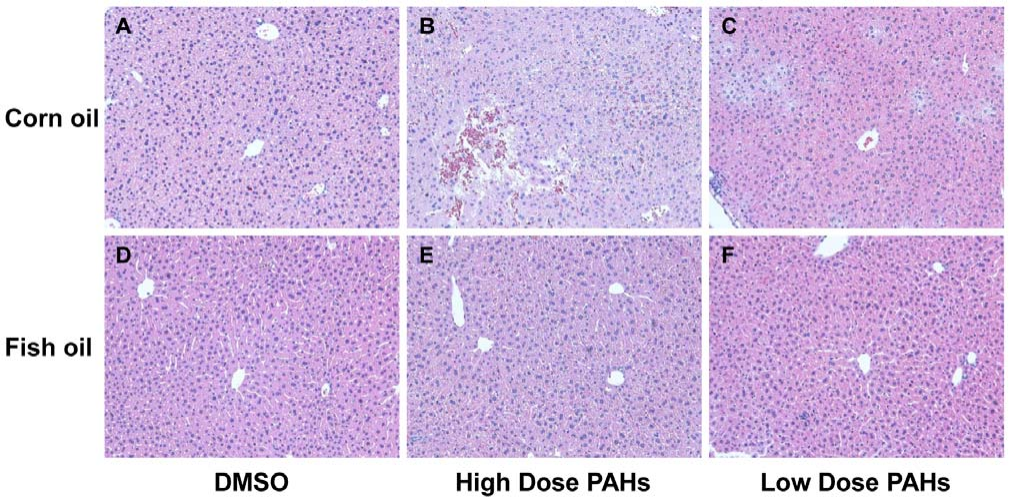
Histological changes in liver of mice treated with carcinogenic PAHs for both FO and CO diet groups. Seven days after treatment of carcinogenic PAH mixture, focal hemorrhage and tissue necrosis were present in dietary CO group of high dose PAHs (Panel B). Congestion in central vein and sinusoids is observed in both PAH doses of CO groups (Panels B and C). Only mild histological changes were displayed in high dose PAHs of dietary FO group (E).

Sections from control mice in both diet groups display normal hepatic architecture and hepatocytes (Figure 4A and 4D). Significant liver damage was observed in CO groups of mice treated with high dose PAHs at 7 d (Figure 4B), but not in FO (Figure 4E). Figure 4B shows that liver tissue consists of focal hemorrhage and necrosis. No sharp boundaries between the cords of hepatocytes and sinusoids are observed (Figure 4B). Histological examinations were also carried out for 1 and 3 d tissue samples. There was no hepatic damage observed in either the FO or CO group. This important histological observation indicated that FO played a hepatoprotective role in early stage.

## Discussion

Complex PAH mixtures are ubiquitous carcinogenic environmental contaminants to which humans are exposed every day. DNA adducts have been proven to be significant biomarkers for carcinogenic PAH exposure in many tissues of human [36], especially in smokers [5]. Previous animal experiments have indicated that levels of carcinogen DNA adducts correlated with tumor incidence in rodents [9], [37]. Epidemiological studies have also shown there were significant positive association between levels of PAH-DNA adducts levels and tumor incidence [7], [8], [38]. Therefore, it is very important to develop approaches that can decrease the formation of DNA adducts and can remove adducted nucleotides and damaged cells with DNA adducts. These strategies are expected to significantly reduce the risk of cancer, especially in high risk populations such as cigarette smokers and others exposed to environmental PAHs.

Our experiments have indicated that mice on a FO diet displayed lower hepatic PAH-DNA adduct levels from 1 through 7 d after PAH treatment compared to CO groups. Significant differences of total adduct levels (Figure 2) between the two diet groups were observed in all time points and doses, except for low PAH dose at 3 d (P = 0.067). BPDE-dG adduct (Figure 3) displayed slightly different trends compared with total DNA adducts. No significant differences of BPDE-dG were observed at 1 d between two diet groups. At 3 d, FO decreased BPDE-dG significantly (P = 0.033) in low PAH dose group only. FO groups displayed significant lower levels of BPDE-dG in both PAH doses at 7 d compared to CO (Figure 3). These results suggested that it may take more time for FO to inhibit formation of BPDE-dG adducts than to decrease levels of other DNA adducts. At the 7 d time point, both levels of PAH-DNA adducts and expression of *Cyp1a1* gene displayed very significant differences between the FO and CO groups. *Gstt1* exhibited similar trend but only low PAH dose groups showed significant difference (Table 5). These observations suggest that FO, with major components as n-3 fatty acids, may act as chemopreventive agents to attenuate carcinogen-DNA adduct formation. Subsequently, it may reduce the risk of cancer of liver in subjects exposed to PAH carcinogens. Many studies have reported that n-3 fatty acids played very important roles in cancer prevention [39], [40]. It was also reported that frequent fresh fish consumption may reduce the risk of lung cancer [41].

In 1985, Wattenberg pointed out [42] that chemopreventive agents can be classified into two major group: blocking agents and suppressing agents. Blocking agents prevent carcinogenic compounds from reacting with critical target sites in the tissue. Suppressing agents act after attack by carcinogens [12], [42], [43]. Some agents have both effects.

In the current study, we found that FO could play a role in detoxifying carcinogenic PAHs by mechanisms entailing induction of phase II enzymes and/or Cyp1a1. This was supported by histological results (Figure 4). The hepatic toxicity by PAHs was obviously attenuated in FO group at 7 d. Therefore, we believe that FO may play a role as a blocking agent in our current study. It is possible that *Cyp1a1* and *Gstt1*genes, and possibly other related genes that we did not analyze this time, may have been involved in the detoxification of PAHs. Although *Cyp1a1* is known to be involved in metabolic activation of PAHs to reactive intermediates, recent studies have suggested that Cyp1a1 also contributes to the detoxification of BaP [44], [45]. In the current study, it is quite possible that induction of Cyp1a1 in part contributed to the decrease of PAH-DNA adduct formation by FO. Glutathione *S*-transferases (GSTs) are a class of phase II enzymes, which can detoxify PAH epoxides as well as quinones, and hydroperoxides by conjugation with gluthathione [46], [47]. The induction of Gsst1 by FO may also have contributed to the decreased PAH-DNA adduct levels in mice fed with FO diet.

Histopathological results showed that high dose of PAHs caused severe liver damage at 7 d after treatment in CO group (Figure 4), but not in FO group. These results suggested that FO played a protective role in liver against PAH toxicity at 7 d time point. These results also support the hypothesis that detoxification of carcinogenic PAHs by FO is one of pathways responsible for the decreased hepatic DNA adducts in these mice.

FO may eliminate adducted nucleotides through increasing apoptosis [16], [48], inhibition of cell proliferation [19]. Although dietary FO may reduce oxidative DNA damage [16], [49], [50] and play a role as antioxidant in certain diseases [50], there is no direct evidence to show that FO can enhance DNA repair activities, as bulky DNA adducts are formed by exposure of chemical carcinogens at early stages, such as at 7 d. It has been reported that n-3 fatty acids decrease cell growth [51] and induce apoptosis in many human cancer cells [40] such as colonic [52], prostate [53], pancreatic [54], pulmonary [19], and breast cancers [55]. We have also reported [14] that retinoic acid, which could be a suppressing agent [42], diminishes BaP-adducts in HepG2 cells partially by inducing apoptosis. However, n-3 fatty acids did not induce significant apoptosis in our current animal experiments. Apoptosis may be a secondary reaction of protection when animals are exposed to chemical toxicants. Therefore, this protective approach may be observed after 7 d of the PAH treatment.

As one of the natural chemopreventive agents, FO containing n-3 fatty acids has received great attention for cancer prevention because of its various health benefits, lack of toxicity and less side effects [10]. Although the contents of FO in diet were quite high (11.5%), it may not be attained in human population. Yam et al [20] reported that 4% FO could also suppress tumor growth and metastasis. In the future studies, long term regimen and lower concentration of FO should be investigated in cancer prevention. As mentioned in the Introduction, natural chemopreventive agents prevent chemical carcinogenesis mainly through three pathways, i.e. detoxification, DNA repair, and apoptosis. Different natural product may have different pathway(s) against chemical carcinogenesis. To increase the efficiency in chemoprevention in high risk population, the combination of natural chemopreventive agents may provide potential and efficient benefits in cancer prevention, especially in chemical carcinogenesis.

In conclusion, our results showed that FO significantly deceased levels of both BPDE-dG and total PAH DNA adducts in the liver of B6C3F1 male mice at 7 d after treatment of carcinogenic PAHs. Also, FO significantly enhanced expression of *Cyp1a1* and *Gstt1* genes (low dose of PAHs) at 7 d after PAH treatment. Histological observation indicated that FO protected liver against PAH toxicity. Further studies could lead to the development of novel strategies (e.g., dietary FO, or combination of chemopreventive agents) in the prevention of human cancers caused by environmental PAHs.

## References

[1] World Health Organization (WHO) website. Cancer.. http://www.who.int/mediacentre/factsheets/fs297/en/.

[2] Jemal A, Siegel R, Xu J, Ward E (2010). Cancer statistics, 2010.. CA Cancer J Clin.

[3] Grant WB (2009). Air pollution in relation to U.S. cancer mortality rates: an ecological study; likely role of carbonaceous aerosols and polycyclic aromatic hydrocarbons.. Anticancer Res.

[4] U.S. EPA Website.. http://cfpub.epa.gov/ncea/iris/index.cfm?fuseaction=iris.showKeywordResults&maxrows=15&startrow=1&textfield=probablehumancarcinogenicPAHs&searchtype=irisdata&x=13&y=16.

[5] Hecht SS (2003). Tobacco carcinogens, their biomarkers and tobacco-induced cancer.. Nat Rev Cancer.

[6] Wogan GN, Hecht SS, Felton JS, Conney AH, Loeb LA (2004). Environmental and chemical carcinogenesis.. Semin Cancer Biol.

[7] Tang D, Phillips DH, Stampfer M, Mooney LA, Hsu Y (2001). Association between carcinogen-DNA adducts in white blood cells and lung cancer risk in the physicians health study.. Cancer Res.

[8] Bak H, Autrup H, Thomsen BL, Tjonneland A, Overvad K (2006). Bulky DNA adducts as risk indicator of lung cancer in a Danish case-cohort study.. Int J Cancer.

[9] Phillips TD (2006). The relationships between levels of DNA adducts and tumor incidence in different tissues of B6C3F1 male mice treated with benzo(a)pyrene and a reconstituted PAH mixture..

[10] Manson MM, Farmer PB, Gescher A, Steward WP (2005). Innovative agents in cancer prevention.. Recent Results Cancer Res.

[11] van Beelen VA, Aarts JM, Reus A, Mooibroek H, Sijtsma L (2006). Differential induction of electrophile-responsive element-regulated genes by n-3 and n-6 polyunsaturated fatty acids.. FEBS Lett.

[12] Wattenberg LW (1992). Inhibition of carcinogenesis by minor dietary constituents.. Cancer Res.

[13] Katiyar SK, Vaid M, van Steeg H, Meeran SM (2010). Green tea polyphenols prevent UV-induced immunosuppression by rapid repair of DNA damage and enhancement of nucleotide excision repair genes.. Cancer Prev Res (Phila Pa).

[14] Zhou GD, Richardson M, Fazili IS, Wang J, Donnelly KC (2010). Role of retinoic acid in the modulation of benzo(a)pyrene-DNA adducts in human hepatoma cells: implications for cancer prevention.. Toxicol Appl Pharmacol.

[15] Velmurugan B, Singh RP, Agarwal R, Agarwal C (2010). Dietary-feeding of grape seed extract prevents azoxymethane-induced colonic aberrant crypt foci formation in fischer 344 rats.. Mol Carcinog.

[16] Hong MY, Bancroft LK, Turner ND, Davidson LA, Murphy ME (2005). Fish oil decreases oxidative DNA damage by enhancing apoptosis in rat colon.. Nutr Cancer.

[17] Fan YY, Ran Q, Toyokuni S, Okazaki Y, Callaway ES (2011). Dietary fish oil promotes colonic apoptosis and mitochondrial proton leak in oxidatively stressed mice.. Cancer Prev Res (Phila).

[18] Gao L, Wang J, Sekhar KR, Yin H, Yared NF (2007). Novel n-3 fatty acid oxidation products activate Nrf2 by destabilizing the association between Keap1 and Cullin3.. J Biol Chem.

[19] Mernitz H, Lian F, Smith DE, Meydani SN, Wang XD (2009). Fish oil supplementation inhibits NNK-induced lung carcinogenesis in the A/J mouse.. Nutr Cancer.

[20] Yam D, Peled A, Shinitzky M (2001). Suppression of tumor growth and metastasis by dietary fish oil combined with vitamins E and C and cisplatin.. Cancer Chemother Pharmacol.

[21] Kang JX, Wang J, Wu L, Kang ZB (2004). Transgenic mice: fat-1 mice convert n-6 to n-3 fatty acids.. Nature.

[22] Weylandt KH, Krause LF, Gomolka B, Chiu CY, Bilal S (2011). Suppressed liver tumorigenesis in fat-1 mice with elevated omega-3 fatty acids is associated with increased omega-3 derived lipid mediators and reduced TNF-{alpha}.. Carcinogenesis.

[23] Lim K, Han C, Dai Y, Shen M, Wu T (2009). Omega-3 polyunsaturated fatty acids inhibit hepatocellular carcinoma cell growth through blocking beta-catenin and cyclooxygenase-2.. Mol Cancer Ther.

[24] Reddy MV, Randerath K (1986). Nuclease P1-mediated enhancement of sensitivity of 32P-postlabeling test for structurally diverse DNA adducts.. Carcinogenesis.

[25] Zhou GD, Hernandez NS, Randerath E, Randerath K (1999). Acute elevation by short-term dietary restriction or food deprivation of type I I-compound levels in rat liver DNA.. Nutr Cancer.

[26] Gupta RC (1984). Nonrandom binding of the carcinogen N-hydroxy-2-acetylaminofluorene to repetitive sequences of rat liver DNA in vivo.. Proc Natl Acad Sci U S A.

[27] Randerath K, Reddy MV, Gupta RC (1981). 32P-labeling test for DNA damage.. Proc Natl Acad Sci U S A.

[28] Zhu H, Cabrera RM, Wlodarczyk BJ, Bozinov D, Wang D (2007). Differentially expressed genes in embryonic cardiac tissues of mice lacking Folr1 gene activity.. BMC Dev Biol.

[29] Zhou GD, Randerath K, Donnelly KC, Jaiswal AK (2004). Effects of NQO1 deficiency on levels of cyclopurines and other oxidative DNA lesions in liver and kidney of young mice.. Int J Cancer.

[30] Randerath K, Randerath E, Zhou GD, Supunpong N, He LY (1999). Genotoxicity of complex PAH mixtures recovered from contaminated lake sediments as assessed by three different methods.. Environ Mol Mutagen.

[31] Chang WC, Chapkin RS, Lupton JR (1997). Predictive value of proliferation, differentiation and apoptosis as intermediate markers for colon tumorigenesis.. Carcinogenesis.

[32] Zhou GD, Popovic N, Lupton JR, Turner ND, Chapkin RS (2005). Tissue-specific attenuation of endogenous DNA I-compounds in rats by carcinogen azoxymethane: possible role of dietary fish oil in colon cancer prevention.. Cancer Epidemiol Biomarkers Prev.

[33] Mabon N, Moorthy B, Randerath E, Randerath K (1996). Monophosphate 32P-postlabeling assay of DNA adducts from 1,2∶3,4-diepoxybutane, the most genotoxic metabolite of 1,3-butadiene: in vitro methodological studies and in vivo dosimetry.. Mutat Res.

[34] Zar JH (2009). Biostatistical Analysis. Fifth edition.

[35] Randerath E, Zhou GD, Donnelly KC, Safe SH, Randerath K (1996). DNA damage induced in mouse tissues by organic wood preserving waste extracts as assayed by 32P-postlabeling.. Arch Toxicol.

[36] Phillips DH (2002). Smoking-related DNA and protein adducts in human tissues.. Carcinogenesis.

[37] Poirier MC, Beland FA (1994). DNA adduct measurements and tumor incidence during chronic carcinogen exposure in rodents.. Environ Health Perspect.

[38] Gunter MJ, Divi RL, Kulldorff M, Vermeulen R, Haverkos KJ (2007). Leukocyte polycyclic aromatic hydrocarbon-DNA adduct formation and colorectal adenoma.. Carcinogenesis.

[39] Rose DP, Connolly JM (1999). Omega-3 fatty acids as cancer chemopreventive agents.. Pharmacol Ther.

[40] Wendel M, Heller AR (2009). Anticancer actions of omega-3 fatty acids–current state and future perspectives.. Anticancer Agents Med Chem.

[41] Takezaki T, Inoue M, Kataoka H, Ikeda S, Yoshida M (2003). Diet and lung cancer risk from a 14-year population-based prospective study in Japan: with special reference to fish consumption.. Nutr Cancer.

[42] Wattenberg LW (1985). Chemoprevention of cancer.. Cancer Res.

[43] Wattenberg LW (1996). Chemoprevention of cancer.. Prev Med.

[44] Uno S, Dalton TP, Derkenne S, Curran CP, Miller ML (2004). Oral exposure to benzo[a]pyrene in the mouse: detoxication by inducible cytochrome P450 is more important than metabolic activation.. Mol Pharmacol.

[45] Uno S, Dalton TP, Dragin N, Curran CP, Derkenne S (2006). Oral benzo[a]pyrene in Cyp1 knockout mouse lines: CYP1A1 important in detoxication, CYP1B1 metabolism required for immune damage independent of total-body burden and clearance rate.. Mol Pharmacol.

[46] Hayes JD, Flanagan JU, Jowsey IR (2005). Glutathione transferases.. Annu Rev Pharmacol Toxicol.

[47] Garte S, Taioli E, Popov T, Kalina I, Sram R (2007). Role of GSTT1 deletion in DNA oxidative damage by exposure to polycyclic aromatic hydrocarbons in humans.. Int J Cancer.

[48] Hong MY, Lupton JR, Morris JS, Wang N, Carroll RJ (2000). Dietary fish oil reduces O6-methylguanine DNA adduct levels in rat colon in part by increasing apoptosis during tumor initiation.. Cancer Epidemiol Biomarkers Prev.

[49] Bancroft LK, Lupton JR, Davidson LA, Taddeo SS, Murphy ME (2003). Dietary fish oil reduces oxidative DNA damage in rat colonocytes.. Free Radic Biol Med.

[50] Mahadik SP, Pillai A, Joshi S, Foster A (2006). Prevention of oxidative stress-mediated neuropathology and improved clinical outcome by adjunctive use of a combination of antioxidants and omega-3 fatty acids in schizophrenia.. Int Rev Psychiatry.

[51] Boudreau MD, Sohn KH, Rhee SH, Lee SW, Hunt JD (2001). Suppression of tumor cell growth both in nude mice and in culture by n-3 polyunsaturated fatty acids: mediation through cyclooxygenase-independent pathways.. Cancer Res.

[52] Pan J, Keffer J, Emami A, Ma X, Lan R (2009). Acrolein-derived DNA adduct formation in human colon cancer cells: its role in apoptosis induction by docosahexaenoic acid.. Chem Res Toxicol.

[53] Berquin IM, Min Y, Wu R, Wu J, Perry D (2007). Modulation of prostate cancer genetic risk by omega-3 and omega-6 fatty acids.. J Clin Invest.

[54] Strouch MJ, Ding Y, Salabat MR, Melstrom LG, Adrian K (2009). A High Omega-3 Fatty Acid Diet Mitigates Murine Pancreatic Precancer Development.. J Surg Res.

[55] Jude S, Roger S, Martel E, Besson P, Richard S (2006). Dietary long-chain omega-3 fatty acids of marine origin: a comparison of their protective effects on coronary heart disease and breast cancers.. Prog Biophys Mol Biol.

